# Diagnostic value of median nerve ultrasonography for screening of carpal tunnel syndrome in hypothyroid patients: A cross-sectional study

**Published:** 2016-04-03

**Authors:** Masoud Mehrpour, Zahra Mirzaasgari, Mohammad Rohani, Mahdi Safdarian

**Affiliations:** 1 Department of Neurology and Stroke Center, Firoozgar General Hospital, Iran University of ‎Medical Sciences, Tehran, Iran; 2 Department of Neurology, Rasoul Akram General Hospital, Iran University of Medical Sciences, ‎Tehran, Iran; 3 Student of Medicine, School of Medicine, Iran University of Medical Sciences, Tehran, Iran

**Keywords:** Carpal Tunnel Syndrome, Ultrasonography, Hypothyroidism

## Abstract

**Background:** Carpal tunnel syndrome (CTS) is a common peripheral entrapment neuropathy in patients with hypothyroid. The diagnosis of CTS is usually clinical and confirmed by electrodiagnostic (EDX) procedures. This study aimed to describe the diagnostic accuracy of high-resolution ultrasonography (US) as an alternative method to nerve conduction study (NCS) for the diagnosis of subclinical CTS in patients with hypothyroidism.

**Methods:** Between April 2013 and November 2014, from the patients with the diagnosis of hypothyroidism referring to the institute of endocrinology and metabolism of Firoozgar Hospital, Tehran, Iran, those who met our inclusion criteria entered this cross-sectional study. The patients divided into two groups of subclinical CTS with the age- and gender-matched control group. US measurements of the median nerve cross-sectional area (CSA) in the CT inlet were compared with the NCS results as the gold standard diagnostic test.

**Results:** A total number of 152 wrists of 76 hypothyroid patients were examined in this study. The mean of median nerve CSA at the tunnel inlet was 9.96 ± 2.20 mm^2^ for the CTS group and 7.08 ± 1.38 mm^2^ for the control group (P < 0.05). 31 wrists (20.4%) were diagnosed as CTS using NCS while US diagnosed 19 wrists (12.5%) as CTS. Using receiver-operating-characteristics analysis, the sensitivity and specificity of US at the diagnosis of CTS were 45.0 and 95.8%, respectively, with a median nerve CSA cutoff point of 9.8 mm^2^. Positive and negative predictive values of US were 87.2 and 85.5%, respectively, with a test accuracy of 85.5%.

**Conclusion:** According to our findings, US has an acceptable diagnostic value to confirm CTS in hypothyroid patients. However, it may not replace NCS due to low sensitivity.

## Introduction

Musculoskeletal disorders are common in patients with hypothyroidism,^1^ and carpal tunnel syndrome (CTS) is the most common compression syndrome in the upper extremities.^2^ The recent American Academy of Orthopaedic Surgeons Clinical Guidelines define CTS as asymptomatic compression neuropathy, which is characterized by decreased median nerve function at the level of wrist accompanying with physiologically increased pressure in the CT. With a prevalence of about 50 per 1000 subjects, the incidence of CTS has been estimated 1-3 per 1000 cases per year in the United States.^3^ Current literature reports a higher prevalence of hypothyroidism and diabetes in patients with CTS. The prevalence of CTS is estimated to be 5.8% in women and 0.6% in men, in the general population^4^ while its prevalence in hypothyroid patients is reported about 28.5%.^1^ A retrospective review of patients who underwent surgery for CTS over a 3-year period by Vashishtha et al.^5^ in the UK showed that CTS is associated with thyroid dysfunction and diabetes.

The British Society for Surgery of the Hand advises screening CTS patients for thyroid and glucose dysfunction before surgery.^5^ Considering the high prevalence of CTS in hypothyroidism, early diagnosis of this disorder to prevent nerve changes is very important. CTS is characterized by typical anatomic changes, the most probable swelling of the median nerve in the proximal part of the CT.^6^^,^^7^ The diagnosis is usually clinical using Tinel’s sign and Phalen’s maneuver and is confirmed by nerve conduction study (NCS).^8^ Although NCS is the method most frequently used in practice to confirm a clinical diagnosis of CTS, it is known to be painful or unpleasant for patients, and false negatives and false positives occur even if the most sensitive methods are used.^2^ In addition, NCS does not provide anatomical information about the nerve or its surroundings that could help in determining its etiology.^9^ Recently, using high-frequency ultrasonography (US) for CTS has emerged as an alternative confirmatory test with a sensitivity of 44-95% and a specificity of about 57-100%.^10^ Various studies^1^^,^^11^^-^^13^ suggested different cut-off points to have the best sensitivity and specificity for median nerve cross-sectional area (CSA) as the most reliable finding for the diagnosis of CTS.^14^

Yet, no study has been conducted to evaluate the diagnostic value of high-frequency US in the diagnosis of CTS in patients with hypothyroidism. To determine whether these findings are reliable and can be used to establish the diagnosis, we aimed to evaluate US as an alternative procedure for diagnosis of subclinical CTS in hypothyroid patients in a cross-sectional study.

## Materials and Methods

This study was approved by the Local Ethics Committee of the Firoozgar Clinical Research Development Center, Iran University of Medical Sciences, Tehran, Iran. Informed consent was taken from the patients before the diagnostic procedures. No invasive method was used in this study and all the diagnostic procedures were harmless. The patients’ information remains confidential and would be used only for analytical study. 

Between April 2013 and November 2014, from the patients with the diagnosis of hypothyroidism referring to the institute of endocrinology and metabolism of Firoozgar Hospital, those who met our inclusion criteria entered the study. The patients with CTS or injection, wrist fractures, median nerve neuropathy and cervical radiculopathy, or polyneuropathy were excluded. In addition, the patients with diseases and conditions associated with CTS including pregnancy, rheumatoid arthritis (RA), diabetes mellitus (DM), renal failure, gout, tenosynovitis, tumors, ganglion cysts, amyloidosis, and median nerve with two or more branches were also excluded from the study. Bilateral wrist US at the CT inlet was done for the included patients using a MyLab™40 - Esaote with an 18 MHz ultrasound probe by an expert in the neurology clinic to measure the median nerve CSA. 

To compare the US measurements with the standard values, NCS was done for all patients as the gold standard test. Other variables such as age, body mass index (BMI), disease duration, wrist circumference, and median nerve CSA were recorded for each patient in the data collection form. The patients were divided into two groups of subclinical CTS and the control group according to the clinical Tinel’s sign and Phalen’s tests, confirmed by NCS. The median nerve CSA at the forearm CT inlet was assessed by an expert investigator blinded to the clinical and NCS data.

The subjects were seated facing the examiner with their arms extended, their wrists on a flat surface, their forearms supine, and their fingers semi-extended. The transverse US of the median nerve was performed at the inlet of the CT. The pisiform bone was used as an anatomical landmark to measure the median nerve CSA at the CT inlet, by tracing a continuous line within the hyperechogenicity boundary of the nerve.

A t-test was used to compare these data with those with previous normal wrists. P < 0.05 was considered as statistically significant. Positive and negative predictive values were calculated using SPSS software (SPSS Inc., Chicago, IL, USA). To compare the accuracy of US, sensitivity and specificity were measured using receiver-operating characteristic curve analysis and other diagnostic test evaluations. Quantitative variables expressed as mean ± standard deviation (SD) and frequency was used for qualitative data.

## Results

**Figure 1:**
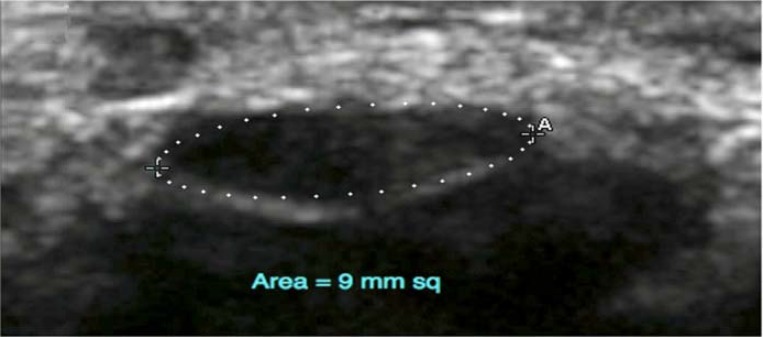
Transverse ultrasonographic image of median nerve (outlined) at level of carpal tunnel (CT) inlet

A total number of 76 patients (152 wrists bilaterally) were recruited in the study including 70 women and 6 men. The mean age of the subjects was 43.1 ± 13.8 years (range from 19 to 81) with a mean BMI of 27.5 ± 3.8 kg/m^2^ (minimum 20 and maximum 45). The mean duration of disorder was 7.5 ± 3.3 years (from 2 to 15). The average wrists surface area was 20.0 ± 1.7 cm^2^ (minimum 16 and maximum of 24), and the average of median nerve CSA at the CT inlet was 7.6 ± 1.9 mm^2^ (minimum of 4 and maximum of 15). About 31 wrists (20.0%) had CTS diagnostic criteria in the NCS, which were considered as sub-clinical CTS. The median nerve CSA was more than 9 mm^2^ in 14 cases (9.0%) (Figure 1). The mean median nerve CSA at the tunnel inlet was 9.96 mm^2^ (SD: 2.2) for the CTS affected wrists and 7.08 mm^2^ (SD: 1.38) for the normal wrists (P < 0.05). The average of median nerve CSA was 8 mm^2^ in left and 7.34 mm^2^ in right wrists. The same parameter was 8 mm^2^ in males and 7.6 mm^2^ in females (The difference was not significant). The average of median nerve CSA seemed also not to be associated with age, BMI, wrist circumference and duration of the disease. According to the NCS findings, 31 wrists (20.4%) were diagnosed as CTS while 19 wrists (12.5%) were diagnosed as CTS by the US. The sensitivity and specificity of US in the diagnosis of CTS was 45.0 and 95.8%, respectively, with a CSA cutoff point of 9.8 mm^2^. Positive and negative predictive values of US were 73.7 and 87.2%, respectively, with a test accuracy of 85.5%.

## Discussion

Although CTS is clinically diagnosed, moderate sensitivity and specificity have been reported for clinical symptoms^15^ and false negative and positives results for NCS.^16^^,^^17^ A some of the CTS symptoms such as paresthesia may appear before nerve fiber changes, which can justify the false negative results of NCS. According to different studies, NCS has a sensitivity of about 56-85% and a specificity of 94% or more in the diagnosis of CTS.^8^ Although semi-invasive, NCS is the gold standard diagnostic procedure for CTS, while comparing to US, the electrical stimuli may not be pleasant for the patient.^3^^,^^4^

In our study, the median nerve US had a sensitivity and specificity of 45% and 95.8% for diagnosis of CTS in patients with hypothyroid. The US measurements of median nerves were found to be increased significantly in patients with CTS when compared with controls, particularly in terms of CSA. As a result, high-frequency US can be used to confirm the diagnosis and due to high positive predictive value, it can be a suitable test to evaluate CTS in the majority of patients with clinical suspicion of CTS, reserving NCS only for with negative US findings. Current literature approves that patients with CTS have a higher prevalence of hypothyroidism and screening helps diagnosing new cases of this condition in this selected group.^17^ A random-effects meta-analysis of the studies not controlling their estimates for any confounder confirmed an association between CTS and hypothyroidism.^18^

In an interesting study by Kolovos and Tsiotas^19^ to establish US examination as a method with at least of the same accuracy with electrodiagnostic (EDX) study, 60 healthy individuals and 30 patients suffering from CTS were scanned. The authors suggested that ratios over the value 1.0 could be considered as a definite diagnosis of CTS. While, ratios up to 0.79 would be surely refers to a healthy wrist and the intermediate ratios between 0.79 and 1.0 refers to a gray zone, which is practically considered healthy.

Recent studies have demonstrated advantages of US in the diagnosis of CTS; however, its role is limited due to lack of adequate data relative to EDX testing. The wide variations of sensitivities and specificities reported in the literature, on the other hand, prevent meaningful analysis of US as either screening or confirmatory test in the diagnosis of CTS.^2^ The meta-analysis by Fowler et al. on 19 articles with a total sample size of 3131 wrists reported the sensitivity and specificity of US for the diagnosis of CTS, 77.6 and 86.8%, respectively.^2^ The study by Kwon et al.^20^ also showed a sensitivity of 66.0% and specificity of 63.0% for median nerve CSA of 10.7 mm^2^ for the US, while the sensitivity and specificity of NCS were 78.0 and 83.0%, respectively. In another study by Mohammadi et al.,^21^ the median nerve CSA at the CT inlet and outlet were examined bilaterally in 82 patients with electrophysiologically confirmed CTS to determine whether high-resolution US can be considered as an alternative diagnostic method to NCS in grading the severity of CT. The differences of the median nerve CSA in mild, moderate and severe CTS were not statistically significant either in the CT inlet or in outlet. According to this study, US of the median nerve CSA had a diagnostic value to confirm or exclude CTS, but could not be used for grading of its severity. In contrast, Azami et al.^22^ prospectively to examined individuals with EDX proven CTS and healthy control subjects to determine the diagnostic value of US compared with NCS. With a sensitivity and specificity of 99.2 and 88.3%, CSA at the tunnel inlet with a threshold of 9.15 mm^2^ had the best diagnostic accuracy. There was reported a significant difference in the median nerve CSA in mild, moderate and severe CTS in this study. In another cross-sectional case-control study, Ghasemi et al.^23^ assessed findings in US in correlation with severity of CTS. According to EDX, patients were classified as mild, moderate, and severe CTS and high-resolution US for CSA measurement at the tunnel inlet was performed for all patients. The mean of the CSA in mild, moderate and severe CTS wrists were 0.12 cm^2^ in, 0.15 and 0.19 cm^2^, respectively. A significant correlation between the median nerve CSA and the severity of CTS impelled the authors claiming that US may serve as a complementary and reliable method in assessing the severity of CTS.

Yazdchi et al.^13^ also studied the sensitivity and specificity of median nerve US in diagnosis of CTS in 90 Iranian patients with clinically suspected CTS. The median nerve CSA at the three levels of the CT could fairly differentiate severe CTS from other cases. Their results suggested that median nerve US cannot replace the NCS because of an overall low sensitivity and specificity.^13^ Dejaco et al.^24^ prospectively studied 135 consecutive patients with diagnosis of CTS in order to compare US measurement of median nerve CSA. They measured CSA using US at five different levels at forearm and wrist. The US of median nerve swelling revealed a good reliability with an intraclass correlation coefficient of 0.90, allowing a fairly reliable diagnosis of CTS.

Kim et al.^25^ studied 187 patients, to determine the criteria for US measurement of the median nerve CSA and differential diagnosis of patients with CTS with or without diabetic polyneuropathy. All the CSAs in this study were larger in the diabetic polyneuropathy group compared with those in the control group. The cutoff value for the CSA at the wrist that yielded the highest sensitivity and specificity was 11.6 mm^2^. They concluded that diagnosing the comorbidity of CTS with diabetic polyneuropathy could be done according to the median nerve CSA at the wrist and the wrist-forearm ratio. In another similar study, Kanikannan et al.^26^ compared the diagnostic accuracy of high-resolution US and electrophysiology in the diagnosis of CTS in patients with CTS and CTS associated with peripheral neuropathy. High-resolution US showed a good correlation with EDX studies in all grades of CTS in these patients with the sensitivity, specificity, positive predictive value and negative predictive values of 76.4, 72.7, 89.5 and 68.0 percent, respectively. They claimed that US can be used as a complementary screening tool to EDX.

Although US is an operator-dependent procedure and should be done by or under supervision of an expert, it may be preferred by patients since it is painless and easily accessible. The high-resolution US allows direct imaging of the involved nerves, in addition to documentation of nerve shape changes that occur in compressive syndromes. US can also diagnose a spectrum of entrapment causes such as tenosynovitis, ganglia, soft-tissue tumors, bone and joint abnormalities, and anomalous muscles. According to our study, US may not replace EDX testing as the most sensitive and specific diagnostic test for CTS diagnosis in hypothyroid patients, but it can be used as the first line confirmatory test as its accuracy was detected 85.5% in our study.

## Conclusion

We conclude that the results of US are reliable, and the diagnosis of CTS in hypothyroid patients can be established based on US findings. Further studies are recommended with wider series to evaluate the ultrasound in CTS of hypothyroid patients and confirm our preliminary results.

## Notes:

**How to cite this article: **Mehrpour M, Mirzaasgari Z, Rohani M, Safdarian M. Diagnostic value of median nerve ultrasonography for screening of carpal tunnel syndrome in hypothyroid patients: A cross-sectional study. Iran J Neurol 2016; 15(2): 70-4.
